# Exploration of the Cytoplasmic Function of Abnormally Fertilized Embryos via Novel Pronuclear-Stage Cytoplasmic Transfer

**DOI:** 10.3390/ijms22168765

**Published:** 2021-08-16

**Authors:** Ayako Fujimine-Sato, Takashi Kuno, Keiko Higashi, Atsushi Sugawara, Hiroaki Hiraga, Aiko Takahashi, Keiko Tanaka, Emi Yokoyama, Naomi Shiga, Zen Watanabe, Nobuo Yaegashi, Masahito Tachibana

**Affiliations:** 1 Department of Obstetrics and Gynecology, Tohoku University Hospital, Sendai 980-8574, Japan; demic0571@gmail.com (A.F.-S.); takashikuno@med.tohoku.ac.jp (T.K.); hkeiko818.fuku@gmail.com (K.H.); atsushi.sugawara.b2@tohoku.ac.jp (A.S.); hiroaki.hiraga.d6@tohoku.ac.jp (H.H.); aiko-takahashi@med.tohoku.ac.jp (A.T.); keiko_des_keiko@yahoo.co.jp (K.T.); emy1@mac.com (E.Y.); naomit@med.tohoku.ac.jp (N.S.); zen.watanabe.e8@tohoku.ac.jp (Z.W.); nobuo.yaegashi.c7@tohoku.ac.jp (N.Y.); 2 Department of Obstetrics and Gynecology, Tohoku University Graduate School of Medicine, Sendai 980-8574, Japan

**Keywords:** cytoplasmic transfer, abnormal fertilization, cytoplasmic deficiency, mitochondria, preimplantation

## Abstract

In regular IVF, a portion of oocytes exhibit abnormal numbers of pronuclei (PN) that is considered as abnormal fertilization, and they are routinely discarded. However, it is known that abnormal ploidy still does not completely abandon embryo development and implantation. To explore the potential of cytoplasm from those abnormally fertilized oocytes, we developed a novel technique for the transfer of large cytoplasm between pronuclear-stage mouse embryos, and assessed its impact. A large volume of cytoplast could be efficiently transferred in the PN stage using a novel two-step method of pronuclear-stage cytoplasmic transfer (PNCT). PNCT revealed the difference in the cytoplasmic function among abnormally fertilized embryos where the cytoplasm of 3PN was developmentally more competent than 1PN, and the supplementing of fresh 3PN cytoplasm restored the impaired developmental potential of postovulatory “aged” oocytes. PNCT-derived embryos harbored significantly higher mitochondrial DNA copies, ATP content, oxygen consumption rate, and total cells. The difference in cytoplasmic function between 3PN and 1PN mouse oocytes probably attributed to the proper activation via sperm and may impact subsequent epigenetic events. These results imply that PNCT may serve as a potential alternative treatment to whole egg donation for patients with age-related recurrent IVF failure.

## 1. Introduction

The cytoplasm of oocytes or embryos is known to harbor cytoplasmic factors, including maternal messenger RNA (mRNA), maternally stored proteins, energy substrates, and mitochondria that will impact the proper completion of meiosis, fertilization, and early preimplantation development of the embryo [1,2,3]. These factors are believed to be indispensable for enabling gamete genome reprogramming to an embryonic or totipotent state, thus important for the erasure of methylation marks on the entire genome by genome-wide demethylation [4]. Further, mitochondria are abundant in the female germline (oocytes), playing an important role in oocyte maturation and subsequent early embryonic development via fertilization, as mitochondrial biogenesis in early embryos is known to be arrested [5].

The significance of cytoplasmic function on preimplantation embryos has been demonstrated as early as the 1980s, where the cancellation of the two-cell block in mouse embryos was shown to be restored by the cytoplasmic transfer of cytoplasm from embryos without cleavage arrest [6]. This demonstrated that the addition or supplementation of the missing cytoplasmic factors can obviously improve the developmental fate of early embryos. Further, several encouraging reports have demonstrated that cytoplasmic transfer/replacement techniques overcame “cytoplasmic deficiency”, “oocyte aging”, and even “gene defect” using different stages of oocytes or embryos in mouse [7,8]. Further, some nuclear transfer techniques, also referred to as mitochondrial replacement therapy (MRT), have been considered to serve as potential germline gene therapy (GGT) applications for inherited mitochondrial diseases [5]. 

Briefly, germinal vesicle transfer (GVT) has been tested with humans and purportedly showed the feasibility and efficacy [9,10]. In 1997, Cohen et al. reported the successful cytoplasmic transfer (CT) in human oocytes, which enabled the production of developmentally competent embryos in patients with compromised oocyte quality, and subsequently reported the live birth of five healthy children in 1998 [11,12]. In 2013, Tachibana et al. demonstrated that developmentally compromised frozen–thawed nonhuman primate oocytes could be rescued by maternal spindle transfer (MST) [13]. Pronuclear transfer (PNT) has also been applied to patients with repeated in vitro fertilization (IVF) cycle failures [14]. Thus, the supplementation or replacement of the cytoplasmic component with a good one could somehow improve the quality of oocytes/embryos irrespective of the stage of oocytes/embryos to be manipulated [5].

The above techniques could potentially provide a benefit for improved assisted reproductive technology (ART) outcomes with options of maintaining the hereditary nuclear DNA, as opposed to the limitations of existing current ART applications, which are confined to the use of donated embryos and oocytes. However, these applications require the donation of fresh-quality oocytes or embryos; thus, the destruction of those is unavoidable. During conventional IVF, a portion of oocytes are known to exhibit abnormal fertilization, which is defined by abnormal numbers of pronuclei (PN), and are routinely discarded. However, in fact, it has been known that some of those embryos could also develop to the blastocyst and subsequently give rise to a successful implantation or a derivation of functional embryonic stem cells, albeit with chromosomal aberration [13,15,16]. In contrast, the transplantation of normal diploid embryos still does not guarantee successful implantation or live birth in preimplantation genetic testing for aneuploidy (PGT-A) cycles, with implantation rates decreasing with maternal age [17]. Thus, chromosomal integrity and cytoplasmic function should be considered independently. Given that the cytoplasm of abnormally fertilized embryos is fully functional and supports embryonic development, abnormally fertilized embryos might be capable of consider as a potential cytoplast donor in future treatment for “cytoplasmic deficiency”. We herein explored the cytoplasmic function of abnormally fertilized embryos. 

## 2. Materials and Methods

### 2.1. Ethical Approval

All animal procedures were performed in accordance with the Tohoku University protocols and ethical guidelines. The approval number is 2019-MdA-069-1.

### 2.2. Embryos and Mice

B6D2F1 (C57BL/6 × DBA/2) mice were used to create embryos via in vitro fertilization (IVF). ICR mice served as host mothers for embryo transfer. Mice were obtained from Japan CHARLES RIVER LABORATORIES JAPAN, INC (Yokohama, Japan) or SLC Inc. (Hamamatsu, Japan).

Eight- to 12-wk-old female mice were superovulated by the intraperitoneal (i.p.) injection of 5–10 IU of pregnant mare serum gonadotropin (PMSG; ASKA Animal Health, Tokyo, Japan), followed by another i.p. injection of 5–10 IU of human chorionic gonadotropin (hCG; ASKA Animal Health, Tokyo, Japan) 48 h later. Oocytes (classified as “fresh” oocytes) were collected from the oviducts of mice at 15–17 h after the injection of hCG and were preincubated in TYH medium (LSI Medience Co., Tokyo, Japan) at 37 °C under 5% CO_2_ in air for 60 min until insemination. In vivo postovulatory aged oocytes (classified as in vivo “aged” oocytes) were recovered from superovulated mice at 22–24 h after administration of hCG. Sperm was collected from the epididymis of 8- to 12-wk-old male mice at the same time as the collection of oocytes. Sperm was incubated in TYH medium at 37 °C under 5% CO_2_ in air for 60 min until insemination. Consecutively, 3 μL of sperm suspension was added to 200 μL of TYH containing cumulus–oocyte complexes (COCs). The COCs were cultured at 37 °C under 5% CO_2_ in air for 3 h. The cumulus cells were washed and denuded. Pronuclear-stage embryos were observed under an inverted microscope (IX73, Olympus, Tokyo, Japan) and then classified to normally (2PN) and abnormally (1PN, 3PN, and more) fertilized, and cultured separately. Two-cell embryos were transferred to a KSOM medium (ARK Resource Co., Kumamoto, Japan) on the second day of insemination and maintained at 37 °C under 5% CO_2_ up to the blastocyst stage. Blastocyst formation and embryo number were visually assessed at 3.5–4.5 days post coitum (dpc). Blastocyst development was assessed based on the numbers of PN-stage embryos or survived PNCT embryos. As a result of IVF with “aged” oocytes, 3PN formation was extremely rare and mostly 1PN (Table S1). Therefore, either IVF with zona-drilled oocytes or two-sperm ICSI were performed to intentionally create 3PN “aged” embryos. For embryo transfer, blastocyst-stage PNCT embryos were transferred to the pseudopregnant ICR female mouse. Three replicated blastocyst transfers were performed and 14, 12, and 12 PNCT embryos were transferred in each. Pups were recovered 21 days after embryo transfer. F1 mice obtained via embryo transfer were weaned after 3 weeks and subsequently mated to produce F2 pups.

### 2.3. Micromanipulation

Pronuclear-stage mouse embryos at 6–10 h after insemination were used for the cytoplasmic transfer. We previously proposed the concept of a technique, called pronuclear-stage cytoplasmic transfer (PNCT), to allow the transplantation of large cytoplast at pronuclear-stage embryos [5]. The developmental process and details of the PNCT procedure can be found in the Results and Supplementary video. Micromanipulations, involving ICSI and PNCT, were performed using an Olympus IX73 microscope equipped with a XYClone^®®^ red-i 20× laser objective (Hamilton Thorne Ltd., Beverly, MA, USA) and a Piezo-driving unit (MB-U; PRIME TECH LTD., Ibaraki, Japan). ICSI was performed in M2 medium according to a previous publication [18]. In order to intentionally create 3PN in an “aged” oocyte, 2 sperms were injected into a single oocyte. During PNCT, manipulation embryos were placed in microdrops consisting of M2 medium (Sigma-Aldrich, Saint Louis, MO, USA) containing cytochalasin B (Sigma-Aldrich, Saint Louis, MO, USA) (10 μg/mL was for PN removal and 5 μg/mL was for PB removal and cytoplast transfer). Cytochalasin B, a cytoskeletal inhibitor, was used to render the cytoplasm and cell membrane less rigid and less prone to lysis. Fusion between the recipient 2PN embryo and the donor cytoplast was induced by a brief exposure (approximately 5 s) to the Hemagglutinating Virus of Japan Envelope (HVJ-E) (GenomONE-CF EX, Ishihara Sangyo Kaisha Ltd., Osaka, Japan) [19,20]. Undiluted HVJ-E stock was prepared according to the manufacturer’s instruction and aliquoting into 5 µL. HVJ-E working media were made with adding 5 µL of M2 media into thawed HVJ-E stock. The success of PNCT (survival rate) was calculated based on the number of surviving and fused embryos per numbers of embryo subjected to PNCT.

### 2.4. Mitochondrial DNA Copy Number Assay

The relative quantification of mtDNA copy number was assessed at the 2-cell stage. In order to verify the effect of the PNCT technique itself, autologous PNCT using “aged” embryos were employed. In brief, each single embryo from either control or PNCT was subjected to whole-genome amplification using a REPLI-g Single Cell Kit (Qiagen, Hilden, Germany) according to the manufacturer’s instruction. Each sample was then analyzed using the Mouse Mitochondrial DNA Copy Number Kit (Detroit R&D Inc., Detroit, MI, USA) in a Fast Real-time PCR system (Applied Biosystems, Foster City, CA, USA). The relative quantification of mtDNA was performed by the 2-ΔΔCt method with β-actin served as the internal control.

### 2.5. Measurement of Adenosine Triphosphate (ATP)

ATP was measured using an IntraCellular ATP kit (TOYO B-Net, Tokyo, Japan). Briefly, blastocysts were rinsed with phosphate-buffered saline (PBS) (Nacalai Tesque, Kyoto, Japan) and plated on a 96-well plate. The absorbance of luciferase was measured using a TriStar2 LB942 microplate reader (Berthold Technologies GmbH, Bad Wildbad, Germany). A standard curve was generated using a 10-fold dilution series of a lysate with a known ATP concentration. The ATP level of each blastocyst was then interpolated from the standard curve.

### 2.6. Measurement of Oxygen Consumption Rate (OCR)

The measurement of OCR was performed according to previous studies using a Chip-sensing Embryo Respiration Monitoring system (CERMs) [21,22]. Briefly, preheated Cook^®®^ Sydney IVF Follicle Flushing Buffer (COOK Medical Australia Pty Ltd., Brisbane, Australia) supplemented with 10% Serum Substitute Supplement (SSS™; Irvine Scientific, Santa Ana, CA, USA) served as the measuring medium. The measuring plate contained a chip sensor and was filled with 2 mL of measuring medium without mineral oil, and then covered to prevent evaporation. The OCR of up to 5 blastocysts was measured automatically except for placing and retrieving the sample in the center of the pit. The measurement of OCR was made outside an incubator for ≤5 min. The sample was retrieved, washed in 4-(2-hydroxyethyl)-1-piperazinyl ethanesulfonic acid (HEPES)-buffered solution, and returned to the culture medium until the next experiment. Details of the equipment architecture and measurement principles were reported elsewhere [21].

### 2.7. Immunocytochemical Procedure and Cell Count

Immunocytochemical procedures were performed as previously described [22]. In brief, embryos were fixed in 4% paraformaldehyde (Wako Pure Chemical Industries, Ltd., Osaka, Japan) and permeabilized with 0.2% Triton X-100 (Sigma-Aldrich, Saint Louis, MO, USA) and 0.1% Tween-20 (Sigma-Aldrich, Saint Louis, MO, USA). Nonspecific reactions were blocked with appropriate normal serum. Embryos were incubated for 40 min in primary antibodies described below, washed 3 times, and exposed to secondary antibodies conjugated with Alexa Fluor (Thermo Fisher Scientific, Waltham, MA, USA) followed by counterstaining with 2 µg/mL of 4′,6-diamidino-2-phenylindole (DAPI; Sigma-Aldrich Corp., St. Louis, MO, USA) for 10 min. Embryos were mounted on microscope slides with a Vector shield mounting medium (Vector Laboratories, Inc, Burlingame, CA, USA) and examined under an epifluorescence microscope (Nikon, Tokyo, Japan) or fluorescence BZ-X800 microscope (Keyence, Tokyo, Japan). Anti-NANOG antibody (ReproCELL Inc., Kanagawa, Japan) was used to detect the inner cell mass (ICM) epiblast (ICM cell number). Anti-H3K4me3 antibody (Millipore, Billerica, MA, USA) was used to detect the trimethylation of histone H3 lysine 4. Both primary antibodies were rabbit polyclonal. All blastocyst cell nuclei were labeled with DAPI (total cell number). Blastocyst cells were counted with NIS-Elements (Nikon, Tokyo, Japan). The H3K4me3 signal was evaluated using a BZ-X800 (Keyence, Tokyo, Japan) automated fluorescent light microscope. BZ-X800 is capable of performing z-stack image acquisition similar to a confocal microscope, and it can also perform automated imaging and quantification procedures. The average epifluorescence signal density of H3K4me3 per nuclear area (Cell/mm^2^) was quantified using Advanced Analysis Software BZ-H4A ver 1.1.2.4 (Keyence, Tokyo, Japan,) under the memorized same setting, according to a previous publication [23]. 

### 2.8. Statistics

All statistical analyses were performed in JMP^®^ 14 (SAS Institute Inc., Cary, NC, USA). All p values were calculated either by using the unpaired two-tailed Student’s t test or one-way ANOVA with the Turkey–Kramer post hoc test. Statistical significance was set at *p* < 0.05.

## 3. Results

### 3.1. Establishment and Verification of Novel Cytoplasmic Transfer

As we intended to explore the cytoplasmic function of abnormally fertilized embryos, it was necessary to develop a reliable technique to isolate and transfer the cytoplasm of PN-stage embryos instead of total replacement, i.e., PNT. We initially performed a single-step manipulation using either a 15 μm- or 30 μm-diameter pipette with a combination of various concentrations of cytoskeletal inhibitors, such as cytochalasin B (CB) or nocodazole. The pipette size was determined based on our previous mouse PNT (15 μm) and mouse blastomere biopsy (30 μm) experiment. A total of 156 pronuclear-stage embryos were used in 10 replicated experiments. We experienced a low survival rate (40.4%) and low blastocyst rate (9.5%), even when using several combinations of pipette sizes and adjusted concentrations of cytoskeletal inhibitors used (Table S2). 

**Figure 1:**
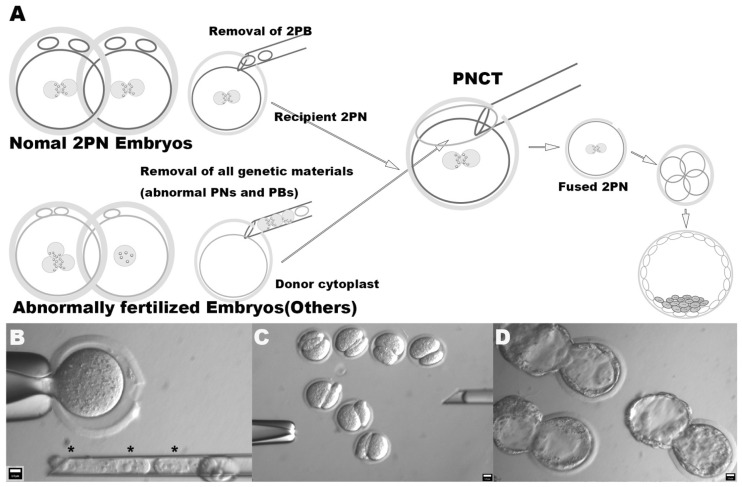
Schematic diagram of PNCT. (**A**) Figure depicts PNCT procedure. Embryos are classified to either normal 2PN embryos or abnormally fertilized embryos. Whereas only 2PBs are removed from the 2PB recipient, all genetic materials are removed from the abnormally fertilized embryo and create the nuclear-free donor cytoplast. Donor cytoplast was isolated followed by brief exposure to HVJ-E. Cytoplast was then transferred into the perivitelline space of recipient 2PN zygotes. (**B**) Figure depicts the removal of 3PNs and 2PBs from 3PN embryos to create nuclear DNA-free donor cytoplast. Asterisks indicate removed 3PNs within pipette. (**C**) Figure depicts post-PNCT embryos prior to fusion occurring. The perivitelline space of recipient 2PN embryos was filled with transferred cytoplast. (**D**) Figure depicts blastocysts derived by PNCT. Original magnifications of (**A**–**C**) are 320×, 200×, and 300×, respectively. * Asterisk indicates enucleated PNs within pipette.

We therefore proceeded with separate micromanipulation steps, including (i) the removal of nuclear materials from both normal (removal of PBs) and abnormal (removal of both PNs and PBs) embryos, and (ii) the transfer of nuclear material-free cytoplast to the perivitelline space of normal 2PN embryos (Figure 1A). The first step was performed using a 15 μm-diameter pipette under a 10 μg/mL concentration of CB (Figure 1B). This step made possible the efficient production of nuclear DNA-free cytoplast with minimal loss of cytoplasm. Further, the removal of PBs from 2PN recipient embryos works prevented the refusion of PBs during fusion of the transferred cytoplast and 2PN cytoplasm by HVJ-E, and also generated more perivitelline space. The second step was performed using a 30 μm-diameter pipette under a 5 μg/mL concentration of CB. This allowed for the efficient transplantation of extremely large cytoplasm, such as transferring half the volume of the cytoplasm from a single abnormally fertilized embryo (Figure 1C). Isolated cytoplast was then briefly exposed to HVJ-E and transferred into the perivitelline space of a 2PN recipient embryo. The two-step method yielded the efficient transplantation of a large amount of cytoplasm while avoiding its lysis and the use of nocodazole, with the reconstructed embryo being stably developed to the blastocyst stage (Figure 1D). Details of the procedure can also be found in the Supplemental video.

**Figure 2:**
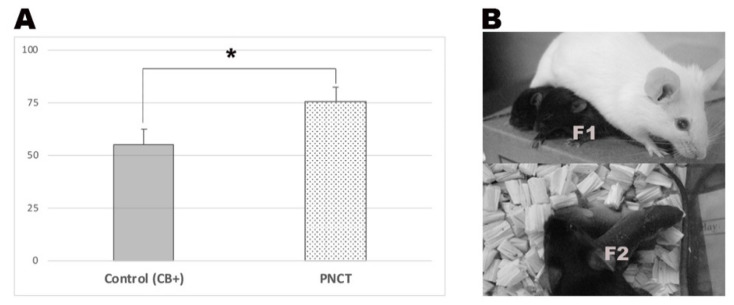
In vitro development of PNCT embryos and PCNT-derived pups. (**A**) Figure depicts the blastocyst rates of PNCT embryos and control embryos. Control 2PN embryos were exposed to a 10 µg/mL concentration of CB for 30 min, referred to as control (CB+). Asterisk indicates statistical significance (*p* < 0.05). (**B**) Upper panel depicts F1 pups derived by PNCT. Lower panel shows F2 pups generated via natural mating of F1.

We next conducted eight replicated PNCT experiments and assessed the developmental competence of the resulting embryos. We created 93 PNCT embryos with the cytoplasm of “fresh” 2PN embryos. In order to assess the only effect of the PNCT procedure, 58 CB exposed “fresh” 2PN embryos served as the control. Whereas 55.2% of control embryos were observed to develop to the blastocyst stage, 75.3% of PNCT embryos developed to the blastocyst stage (*p* = 0.036) (Figure 2). Further, we performed three embryo transfers of blastocyst-stage PCNT embryos and recovered fertile offspring (F1) from one out of three embryo transfers (Figure 2, upper panel and lower panel).

### 3.2. Exploration of Impact of Transfer of Abnormally Fertilized Cytoplast to Post-Ovulatory “Aged” Mouse Embryos by Pronuclear-Stage Cytoplasmic Transfer

It is known that reproductive aging is closely correlated to impaired embryo development. We therefore used post-ovulatory “aged” oocytes, which has been used for studying oocyte aging [24,25,26,27], as a reproductive aging model. Indeed, the developmental ability of “aged” 2PN embryos to reach the blastocyst stage (60.5%) was found to be significantly compromised compared with that of nonmanipulated standard “fresh” 2PN embryos (85.7%) (*p* = 0.0307) (Table 1). Using this model, we next explored the cytoplasmic function of abnormally fertilized embryos by supplementing the cytoplast obtained from abnormally fertilized embryos on the development of mouse “aged” 2PN embryos in vitro. Accordingly, we performed 8 and 11 replicated PNCT experiments using the cytoplasm of “aged” and “fresh” 1PN embryos, respectively. In the case of the “aged” cytoplasm used as the cytoplast donor, the transplanted cytoplasm used came from the same individual; thus, it was referred to as autologous (a)PNCT. In contrast, the procedure in which the cytoplasm of a 1PN embryo obtained from a standard “fresh” IVF procedure was transferred to an “aged” 2PN embryo, it was called heterologous (h)PNCT. We initially explored the cytoplasmic function of 1PN embryos derived from “aged” oocytes and “fresh” oocytes. The transplantation of 1PN cytoplasm did not result in improved in vitro development of “aged” 2PN embryos irrespective of the origin of donors, both “aged” and “fresh”, (aPNCT, 39.0%; and hPNCT, 30.0%; respectively, Table 1). Both aPNCT and hPNCT using “aged” (*p* = 0.0012) and “fresh” (*p* = 0.0012) 1PN cytoplasm were demonstrated to even be deleterious to the development of “aged” 2PN embryos.

We next explored the impact of PNCT using 3PN embryos. We performed 7 and 11 replicated PNCT procedures with “aged” and “fresh” 3PN embryos, respectively. The blastocyst rates of aPNCT and hPNCT with the 3PN cytoplasm of “aged” 2PN embryos “fresh” were 66.3% and 81%, respectively (Table 1). Among the PNCT procedures, both aPNCT and hPNCT using “aged” and “fresh” 3PN were shown to lead to a significantly higher blastocyst rate compared with both aPNCT and hPNCT with 1PN embryos (*p* < 0.05) (Table 1). While aPNCT with “aged” 3PN was not shown to be statistically significant, the developmental ability of embryos to reach the blastocyst stage in hPNCT with “fresh” 3PN was demonstrated to be similar to that of nonmanipulated “fresh” 2PN embryos (85.7%) (*p* = 0.3689) and was higher than that of nonmanipulated “aged” 2PN embryos (60.5%) (*p* = 0.0451) (Table 1).

### 3.3. Verification of Physical Impact of Pronuclear-Stage Cytoplasmic Transfer on Cytoplasmic Function of Mouse Embryos

**Figure 3:**
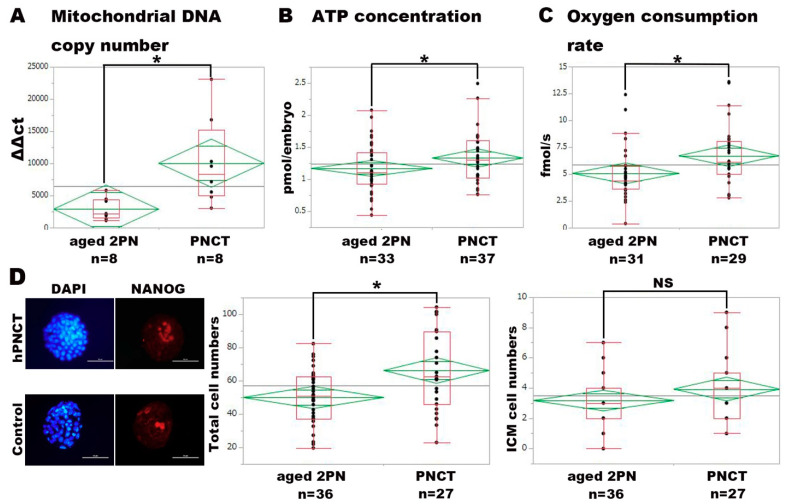
Analyses of mitochondrial DNA copy number, ATP content, oxygen consumption rate, and cell counting for PNCT embryos. (**A**) Figure depicts the relative quantification of mitochondrial DNA copies. PNCT embryos were harvested at the 2-cell stage for both groups. (**B**) ATP concentration of blastocyst-stage embryos is shown. (**C**) Oxygen consumption rate measured by CERMs in the blastocyst-stage embryos is shown. (**D**) Left panel depicts immunohistochemistry against NANOG (for ICM) and DAPI (for total cells). ATP, OCR measurement, and cell counting were performed using blastocysts obtained by PNCT with “fresh” 3PN. White bar indicates 100 µm. Middle panel depicts total cell numbers. Right panel depicts ICM numbers. Asterisk indicates statistical significance (*p* < 0.05). NS indicates statistically not significant.

We next explored the physical effect of performing PNCT-reconstructed embryos compared with nonmanipulated “aged” 2PN embryos, served as the control. Although PNCT was shown to visibly introduce a sufficient amount of cytoplasm, we initially confirmed whether PNCT indeed increased the amounts of mitochondria in reconstructed embryos. The relative quantification of the mtDNA copy number at the two-cell stage revealed a significant increase in mtDNA copies in PNCT embryos compared with controls (*p* = 0.0117) (Figure 3A). We examined the effect of PNCT on mitochondrial function, such as ATP production and oxygen consumption rate (OCR), as a consequence of the increased amounts of mitochondria. The mean ATP concentrations of the PNCT blastocysts using “fresh” 3PN and controls were 0.63 ± 0.044 and 0.47 ± 0.047 pmol/embryo, respectively. The mean OCR of the PNCT blastocysts and the controls were 6.71 ± 0.499 and 5.07 ± 0.483 fmol/s, respectively. Both the contents of ATP and OCR were shown to be significantly increased in PNCT blastocysts compared with controls (ATP, *p* = 0.017; OCR, *p* = 0.026) (Figure 3B,C).

We next aimed to verify the quality of blastocysts derived by PNCT using immunohistochemistry. Blastocysts of “aged” 2PN served as the control. Both blastocysts derived from PNCT and the control were labeled with Nanog for ICM of epiblast and with DAPI for all nuclei, respectively (Figure 3D, left panel). ICM and total cell numbers for the PNCT and controls were 3.9 ± 0.39 and 66.2 ± 3.9, and 3.2 ± 0.34 and 50.0 ± 3.34, respectively. While ICM numbers were observed to be similar between groups, total cell numbers were shown to be significantly increased in PNCT blastocysts compared with controls (*p* = 0.045) (Figure 3D, mid and right).

### 3.4. Differences in Global Methylation of Abnormally Fertilized Mouse Embryos

**Figure 4:**
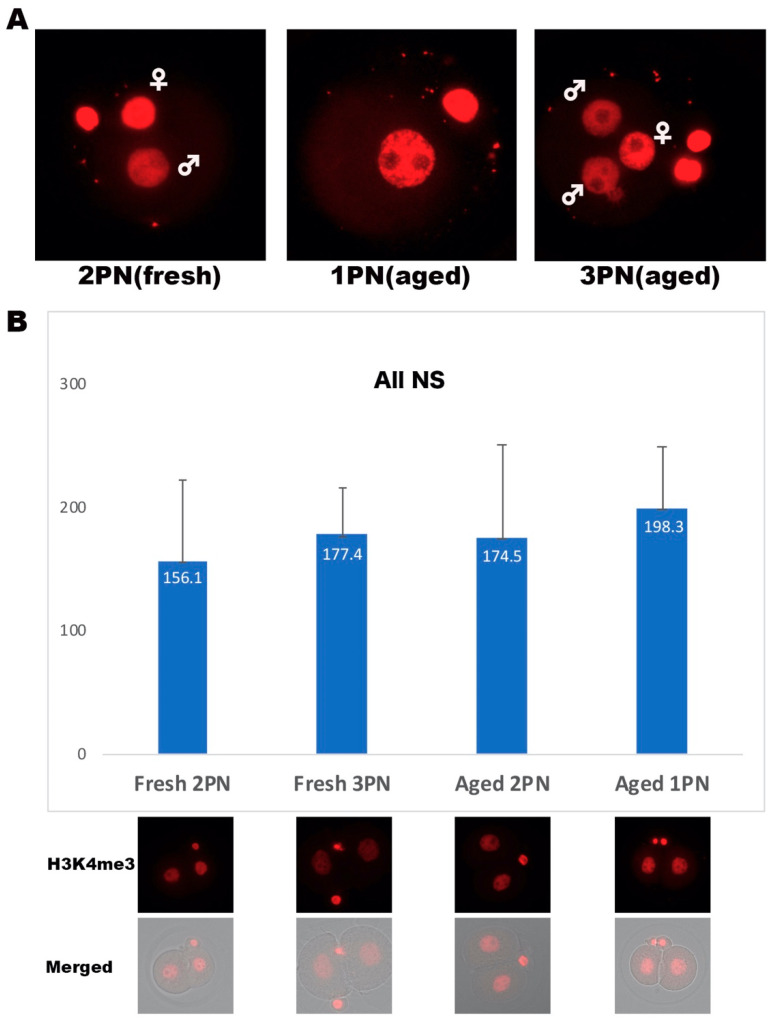
Global methylation analysis by trimethylation of histone H3 lysine 4 immunostaining. (**A**) Figures depict immunostaining of antibody against H3K4me3 in “fresh” 2PN, “aged” 1PN, and “aged” 3PN embryos. ♂ and ♀ indicate male and female pronuclei, respectively. Images were captured using 40× objective. (**B**) Signal intensity of H3K4me3 in 2-cell embryos derived from “fresh” 2PN, “fresh” 3PN, “aged” 2PN, and “aged” 1PN are shown. Bottom images show 2-cell-stage embryos from each origin. NS indicates statistically not significant.

As the above-described PNCT procedures revealed differences in the cytoplasmic function among abnormally fertilized embryos, we looked into the differences in the global demethylation status of normal and abnormally fertilized embryos. We stained both PN-stage and two-cell-stage embryos of various PNs with antibodies against H3K4me3. PN-stage and two-cell stage embryos were collected at 9 and 26 h after insemination, respectively. Male pronuclei were distinguishable by the weak intensity of the H3K4me3 fluorescent signal in 2PN and 3PN embryos, indicating sperm contribution (Figure 4A). We also quantified the fluorescent signal of the H3K4me3 in two-cell embryos derived from “fresh” 2PN (*n* = 10) and 3PN (*n* = 10) embryos, and two-cell embryos derived from “aged” 2PN (*n* = 4) and 1PN (*n* = 12). The mean intensity signal of H3K4me3 for “fresh” 2PN, “fresh” 3PN, “aged” 2PN, and “aged” 1PN were 156.1 ± 65.1, 177.4 ± 37.6, 174.5 ± 75.7, and 198.3 ± 50.2, respectively. The residue signal of H3K4me3 was slightly higher in two-cell embryos derived from “aged” 1PN, compared to others, albeit with no statistical significance (Figure 4B).

## 4. Discussion

In the present study, we initially developed and demonstrated a novel cytoplasmic transfer technique, termed PNCT, which was shown to be feasible, yielding viable embryos in a two-step process. We found that the cytoplasm of 3PN was developmentally competent compared to that of 1PN, irrespective of “aged” or “fresh” oocytes. Further, we demonstrated that the cytoplasmic function of developmentally compromised post-ovulatory “aged” oocytes can be restored when hPNCT was performed with “fresh” 3PN cytoplast. In addition, PNCT was shown to have an impact on mitochondrial function such as ATP production and higher OCR through the increased mtDNA copy number, with the generated blastocysts consisting of large cell numbers. The result of H3K4me3 staining evoked a speculation that functional differences in the cytoplasm between 1PN and 3PN might be the difference in progression of the reprogramming process due to the consequence of with or without proper activation via sperm.

Manipulation of the PN-stage embryo requires several combinations of cytoskeletal inhibitors, such as cytochalasin B (CB), nocodazole, or Colcemid, for making the embryos less prone to lysis at the step of pronucleus isolation/removal [28,29]. An early study showed that a proper concentration of both cytochalasin B and nocodazole might not be detrimental to mouse embryo development [30]. Further, the use of CB during embryo manipulation has been proven to be safe by the live birth of both human infants and nonhuman primate offspring [13,20,31]. However, a viable pregnancy has been achieved by the avoidance of microtubule depolymerization agents, nocodazole, in human PNT [14]. We therefore avoided the use of nocodazole by adjusting the proper concentration of CB and used a combination of two different sizes of micro-pipettes. The two-step method allowed for the efficient transplantation of an extremely large cytoplast (almost half cytoplasm of PN-stage embryo). Unlike other cytoplasmic transfer procedures, PNCT was demonstrated to visibly introduce a large amount of cytoplasm, further indicated by the increased mtDNA copy number in the PNCT embryos. As we demonstrated, PNCT enabled the exploration of the cytoplasmic function of abnormally fertilized embryos, and revealed that the supplementing of the cytoplasm of “fresh” 3PN could restore developmentally compromised “aged” 2PN embryos with increased mitochondrial activity and blastocyst quality. As PNCT resulted in a viable pregnancy, as well as confirmed future fertility, it might serve as a potential treatment for IVF patients with cytoplasmic deficiency. However, we noticed that PNCT embryos showed a slight delay in the development to the blastocyst stage, with the majority of them reaching the fully expanded blastocyst stage by 4, or even 4.5 dpc. Further, the two-cell embryo transfer did not work, probably attributed to a synchronization issue between the embryo stage and host mother. Two live pups were recovered from only one out of three blastocyst-stage embryo transfers, and the other two resulted in either cannibalism or neglect of offspring. We have not yet clarified the factors involved in this slow development and low yield of pups, but assumed that either the technical, the residue of CB, or the timing of manipulation might have affected it. Indeed, the timing has been shown to be important in PNT, and the early PNT showed improved blastocyst rate [7,32]. The PNCT process also requires HVJ-E for the effective fusion of the 2PN recipient embryo and cytoplast. Based on previous studies, the brief exposure of oocytes to these reagents appeared to be nondetrimental [13,20,33,34]. There might be another technical criticism, in that the transplantation of such a large cytoplasm might be nearly impossible in humans due to a relatively small perivitelline space. However, PNCT in human would still warrant a sufficient cytoplast transfer compared with the direct injection technique of reported CT, where only small volumes (1–5%) of donor cytoplasm were able to inject, and some of the derived infants were not found to harbor mitochondrial DNA from donors [11,12,35]. Alternatively, unlike GVT, MST, and CT, PNCT does not contribute to meiosis I and II; the expected effect is restricted to mitotic division during the post-zygotic cleavage [5].

In the clinical IVF program in humans, normal fertilization is defined by the presence of 2PN (male pronucleus: mPN and female pronucleus: fPN) and two polar bodies (first and second polar bodies). However, it has been reported that healthy babies have been born via the transfer of 1PN embryos [36,37], and thus, those could be transferred in patients in the absence of suitable alternatives in clinical IVF [16,38]. However, embryos with 3PN are thought to be triploid and are usually not chosen for transfer in both IVF and ICSI cycles. In contrast to human clinical IVF, fertilization in mouse IVF is usually defined by the cleavage to two-cell-stage embryos. We observed that 1PN mouse embryos were developmentally compromised compared with 3PN and 2PN embryos, irrespective of the origin of embryos from “fresh” or “aged” (Table S1). This finding indicated that mouse 1PN embryos might be predominantly parthenogenetically activated due to spontaneous exit from MII, that is, degradation of the maturation promoting factor. In fact, it was suggested that the absence of sperm incorporation led to postovulatory aging and mediated spontaneous exit from MII. This observation was found to be consistent in across-mammalian species, including mouse [39], rat [40], porcine [41], bovine [42], and human [43,44]. Thus, it is speculated that the main difference of 1PN and 3PN embryos is the proper cytoplasmic activation via sperm. The natural stimuli are known to be brought by changes in osmolality, pH, and temperature [45], and thus have been reported to be extremely inefficient compared to that of sperm factor-derived or various artificial stimulations, such as electroporation, calcium ionophore, and strontium [46]. Further, appropriate oocyte activation is considered to be an indispensable element not only for subsequent embryo development, but also for epigenetic reprogramming of the gamete genome [47]. In early preimplantation embryos, the erasure of DNA methylation marks is known to be an essential process for the reprogramming of both gamete genomes and proper transition of maternal to embryonic gene expression [42,48,49]. The active removal of broad H3K4me3 domains has been shown to be required for normal zygotic genome activation and is important for early embryo development [48]. Our results suggested that the slightly high residue of H3K4Me3 in the two-cell originating from 1PNs “aged” compared to “fresh” 2PN, indicating a delay in global demethylation. This observation indicated the hypothesis that an affected reprogramming process or improper maternal–zygotic transition might be the consequence of an inappropriate cytoplast activation due to the lack of sperm contribution. Despite the cytoplasmic volume being important for the nucleus reprogramming and developmental competence of embryos [50], proper progress of the post-fertilization cytoplasmic event may also be crucial to support subsequent embryo development. However, further study is needed to clarify the mechanism responsible for the difference in cytoplasmic function among abnormally fertilized embryos and, thus, the biological marker indicating cytoplasmic function.

The use of clinically discarded embryos for cytoplasmic transfer is not a completely new idea. In 1999, Huang et al. reported the birth of four healthy infants via CT using 3PN cytoplasm from an unrelated couple undergoing conventional IVF [35]. However, the amount of injected cytoplasm was unknown. In 2010, Craven et al. demonstrated the use of PNT with abnormally fertilized (1PN or 3PN) human embryos aiming to develop a germline gene therapy (GGT) for inherited mitochondrial diseases [28]. Although PNT using abnormally fertilized human zygotes was reported to result in efficient replacement of mtDNA, the rate of blastocyst development of human PNT with abnormally fertilized embryos was shown to be only 8.3%. Further, the PNT efficacy by distinguishing the type of donors, 1PN or 3PN, was unclear. In the present study, we verified an increased cytoplasmic component via PNCT by mtDNA copy number, as well as indicated the origin of abnormally fertilized cytoplasm.

While the cytoplasmic transfer technique might be expected to serve as a potential alterative treatment to egg donation, the introduction of foreign mitochondria may be of concern. The use of heterologous cytoplasm inevitably results in mtDNA heteroplasmy, and it might have a negative impact on the interaction between the nuclear and mitochondrial genomes, metabolic function, and epigenetics [51,52,53,54]. However, a follow-up study of rhesus monkey offspring generated by MST using genetically distant rhesus macaques demonstrated healthy growth with normal reproductive fitness [55]. Further, a recent encouraged clinical trial of human MST with recurrent IVF failure patients in Greece already demonstrated the birth of multiple babies with unrelated donor cytoplasm (https://www.iolife.eu/en/us/mst-research/, accessed date 27 May 2021).

The PCNT effect could be classified by the type of donor, that is, the use of the patient’s own abnormally fertilized embryo is referred to as the autologous (a)PNCT, or that of the donor cytoplast donated from others is referred the heterologous (h)PNCT. Whereas (h)PNCT is known to artificially generate heteroplasmic embryos, (a)PNCT is not, owing to its autologous cytoplasmic transplantation. In contrast, in the case the donor cytoplasm is from a young patient, (h)PNCT might hold not only its quantitative but also its qualitative effect. Though (h)PNCT requires the destruction of embryos from others, it might be more ethically acceptable compared with the alternative cytoplasmic transfer/replacement techniques exploiting discarded embryos.

We herein demonstrated the feasibility of novel cytoplasmic transfer, termed PNCT. Using PNCT, we explored the difference of cytoplasmic function among abnormally fertilized embryos, and also demonstrated the potential usage of PNCT for the restoration of impaired cytoplasmic function. In the present study, we could only demonstrate that (h)PNCT with 3PN embryos showed a complementing effect on the in vitro development of mouse “aged” embryos. However, the current observation in mice may not be fully applied to humans. While additional study is desirable to clarify the mechanisms responsible for the difference in cytoplasmic factors among abnormally fertilized embryos, our technique might offer a new ethically acceptable reproductive option aiming to improve the cytoplasmic function due to aging. As biological features are different in species, the study in humans is indispensable to verify the true biological effect of PNCT. In addition, the results of current clinical trials of both the mitochondrial replacement therapy (MRT) for the inherited mitochondrial disease in UK and the MST for recurrent IVF failure in Greece, need to be closely monitored when (h)PNCT is used because of its germline modification.

## Figures and Tables

**Table 1:** In vitro development of nonmanipulated and PNCT embryos.

	Rep #	Recipient Embryo	Donor Cytoplast	# of Embryos Used	2-Cell (%)	Blastocysts (%)
Aged 2PN	10	NA	NA	223	220 (98.7)	135 (60.5) ^a^
Fresh 2PN	10	NA	NA	300	294(98.0)	257(85.7) ^b^
PNCT with 1PN	8	“aged” 2PN	“aged” 1PN (aPNCT)	123	101(82.1) ^†^	48(39.0) ^c^^†^
	11		“fresh” 1PN (hPNCT)	153	136 (88.9)	46 (30.0) ^c^^†^
PNCT with 3PN	7		“aged” 3PN (aPNCT)	83	71(85.5)	55(66.3) ^‡^
	11		“fresh” 3PN (hPNCT)	142	139 (97.9) ^‡^	115 (81.0) ^b^^‡^

Different alphabetical superscript indicates statistical significance (*p* < 0.05). Between dagger and double dagger indicate statistical significance (*p* < 0.05). aPNCT refers to an autologous cytoplasmic transfer originated from post-ovulatory aged oocytes in same cycle. hPNCT refers to a heterologous cytoplasmic transfer originated from fresh standard IVF embryos.

## Data Availability

Not applicable.

## Supplementary Materials

Supplementary materials can be found at https://www.mdpi.com/article/10.3390/ijms22168765/s1.
