# Supplementary material for: Exploration of the Cytoplasmic Function of Abnormally Fertilized Embryos via Novel Pronuclear-Stage Cytoplasmic Transfer
Source: Int J Mol Sci. 2021 Aug 16;22(16):8765. doi: 10.3390/ijms22168765 (PMC8395835; doi:10.3390/ijms22168765)
Supplement: Supplementary file 1 [file ijms-22-08765-s001.zip › ijms-1313863-proof done supplementary.pdf]

Table S1. In vitro development of mouse IVF embryos with "fresh" and "aged" oocytes.

	Rep #	# of embryos	2-cell (%)	Blastocysts (%)
Fresh 2PN	10	300	294 (98.0)	257 (85.7) [a]
Fresh 1PN	1	13	13 (100.0)	1 (7.7) [na]
Fresh 3PN	3	18	17 (94.4)	12 (66.7) [b]
Aged 2PN	10	223	220 (98.7)	135 (60.5) †
Aged 1PN	9	211	200 (94.8)	21 (10.0) ‡
Aged 3PN	8	51	49 (96.1)	22 (43.1) †

Different superscript indicates statistical significance ($p<0.05$)

Between dagger and double dagger indicate statistical significance ($p < 0.05$)

na: statistics was not applicable

Table S2. Pronuclear stage cytoplasmic transfer using single step manipulation

Exp	# of PN embryos used	Pipette ϕ (μm)*	Cytoskeletal inhibitor(μg/ml)*	# of embryos survived after PNCT	# of embryos cleaved on day 2	Blastocyst
1	10	15	Nocodazol(0.2)+CB(5)	2	2	0
2	10	15	Nocodazol(0.2)+CB(5)	3	3	0
3	10	15	Nocodazol(0.2)+CB(5)	0	0	0
4	20	30	CB(10)	14	8	1
5	20	30	CB(10)	12	11	3
6	10	15	CB(10)	7	5	0
7	16	15	CB(10)	9	8	2
8	20	15	CB(5)	7	0	0
9	20	15	CB(5)	4	0	0
10	20	30	CB(5)	5	0	0
Total	156	NA	NA	63(40.4%)	37(58.7%)	6(9.5%)

CB: Cytochalasin B

* All micromanipulation steps including enucleation of pronucleus, polar bodies, and transfer of large cytoplast were performed in single setting of pipette size and cytoskeletal inhibitor in each experiment.
